# Effect of Career Shocks on Nurses’ Job Performance: Psychological Resilience as a Key Moderator

**DOI:** 10.1155/jonm/8404363

**Published:** 2026-05-18

**Authors:** Na Zhang, Xiang Sun, Xing Bu, Zhen Xu

**Affiliations:** ^1^ School of Business, Beijing Information Science and Technology University, Beijing, China, bistu.edu.cn

**Keywords:** career shocks, job performance, nurses, psychological resilience

## Abstract

**Background:**

In the digital era, the nursing workplace has experienced profound changes, exposing nurses to complex career shocks. Existing professional studies often overemphasize individual agency in career development, neglecting how environmental stressors such as career shocks influence job performance through psychological mechanisms.

**Aim:**

Grounded in the job demands–resources (JD‐R) model, this research examines how positive and negative career shocks differently influence the job performance of nurses, paying particular attention to the moderating effect of psychological resilience.

**Methods:**

This research employs a cross‐sectional quantitative design. A total of 385 nurses were recruited through convenience sampling. Data were collected using questionnaires, and structural equation modeling (SEM) was utilized to analyze the relationships among the variables via Mplus.

**Results:**

The findings show that both positive and negative career shocks have a positive relationship with nurses’ job performance. Psychological resilience demonstrated a significant positive moderating effect on the relationship between positive career shocks and nurses’ job performance, amplifying the beneficial effects of positive shocks; however, it showed no significant moderating effect on the link between negative career shocks and nurses’ job performance.

**Conclusion:**

This study elucidates the influence pathways and mechanisms of positive and negative career shocks on nurses’ job performance, thereby contributing to the advancement of nursing management research.

## 1. Introduction

In today’s variability, uncertainty, complexity, and ambiguity (VUCA) era [1], uncertain factors in the external environment have emerged endlessly and are difficult to predict. This significantly increases the likelihood of employees suffering career shocks [2, 3]. Since the outbreak of COVID‐19, the nursing profession has become increasingly prominent as a core pillar of the healthcare system. As frontline guardians of public health, nurses have long been engaged in clinical practice, and their work is characterized by high risk, high pressure, and intensive collaboration, exposing them to more frequent and impactful career shocks [4]. One prominent challenge currently facing the nursing profession is unstable job performance among nurses. In complex clinical settings such as emergency treatment, doctor–patient communication, and cross‐departmental collaboration, nurses are highly vulnerable to career shocks, including conflicts with patients, staffing shortages, and excessive workloads. These events directly impair the standardization of nursing practice and teamwork efficiency, resulting in performance fluctuations, which not only compromise the quality of nursing care but also pose potential risks to medical safety.

More critically, the acceleration of population aging and the shortage of nurse supply have formed dual pressures, further amplifying the negative impact of career shocks. It is estimated that by 2050, the population over 60 years old in China will reach 478.8 million, and the demand for chronic disease care and disability care brought by aging will continue to surge [5]. However, existing nursing human resources are facing serious turnover risks, with studies showing that 78.3% of nurses have strong or very strong turnover intentions caused by brain drain and shortage pressures [6]. These issues have become practical problems that urgently need to be solved in nursing career management.

Previous career research has emphasized the important role of personal agency in career management [7, 8] but paid little attention to environmental factors such as career shocks [2]. In this context, the study of career shocks has become an even more popular topic in industry and academia. Thus, it is essential to thoroughly investigate the effect of career shocks on nurses’ careers, work behavior, and outcomes. This exploration will contribute to a broader understanding of the influence of career shocks within the nursing field.

Moreover, job performance, as a key focus of organizations, is also a typical work product extensively considered by scholars [9]. A career shock refers to a significant and disruptive occurrence primarily influenced by circumstances outside the individual’s control, prompting profound reflection on their professional life [10]. Consequently, unexpected events in a career significantly influence the development of career paths, along with the job performance and work attitudes of nurses.

According to different levels of psychological valence, career shocks can be categorized into two distinct types: positive career shocks and negative ones [11]. Existing studies have conducted empirical research on unilateral negative career shock [12, 13]. There is a lack of comprehensive empirical investigation on both positive and negative types of career shocks. Furthermore, several researchers have suggested that it is essential to differentiate among the various types, as each can exert unique effects [14]. Consequently, examining how different categories of career shocks influence nurses’ job performance is vital.

Career shocks influence work outcomes by forcing individuals to reallocate and mobilize their career resources [11, 15]. Job performance, a key organizational concern, represents a typical work outcome that has been widely examined by scholars [9, 16]. Given that job performance is inherently multidimensional, we adopt the performance framework for healthcare professionals proposed by Zhang [17] and divide it into two core dimensions: task performance and contextual performance.

In nursing, task performance encompasses core clinical duties explicitly prescribed in job descriptions—such as direct patient care, medication administration, and treatment protocol adherence—whereas contextual performance reflects discretionary behaviors that facilitate collective functioning, including interdisciplinary collaboration, smooth shift handovers, and proactive information sharing [17]. This distinction is particularly critical for nursing management because nursing work is characterized by high interdependence: Patient safety hinges not only on individual clinical competence but equally on seamless coordination across shifts and disciplines. Moreover, career shocks may trigger asymmetric resource reallocation, potentially causing nurses to prioritize technically mandated duties while withdrawing from voluntary collaborative behaviors, or vice versa. Therefore, examining these two dimensions separately allows for a more precise analysis of how career shocks affect nurses’ job performance.

Meanwhile, existing research has documented the paradoxical effects of career shocks, whereby negative shocks may lead to positive outcomes and positive shocks may result in negative consequences [2, 18, 19]. Consistent with the high‐risk, high‐interaction nature of nursing work, negative career shocks mainly stem from adverse events such as doctor–patient conflicts and occupational exposure, whereas positive career shocks arise from positive experiences including patient recognition and professional skill development, which differ substantially in their underlying mechanisms. Therefore, to clearly reveal the mechanism through which career shocks influence job performance, it is necessary to classify both career shocks and job performance into distinct dimensions and explore their differentiated impact pathways.

As research on the job demands–resources (JD‐R) model deepens, scholars have gradually extended the scope of job resources from traditional aspects to include psychological resources, relational resources, and capability resources possessed by individuals. These studies particularly emphasize the profound impact of psychological resources on individuals’ behaviors [20]. Psychological resilience, a key type of psychological capital, centers on one’s ability to cope with challenges in a constructive way [21]. It helps individuals stay emotionally stable, maintain high job performance, and boost continuous personal growth when facing job stress [22].

Moreover, previous research on the boundary of career shocks has mostly approached it from the perspectives of job embeddedness [23], career stage [24], and affective disposition [25]. There is little in‐depth exploration of the boundary role of individual characteristics [4]. Among numerous individual characteristics, psychological resilience is a key factor. It equips an individual with the ability to cope effectively in stressful situations [26]. Nevertheless, the current understanding of how psychological resilience influences the dynamics between diverse career shocks and the job performance of nurses is still ambiguous. It will enrich the research on career shocks in nursing and further elaborate on the concept of job resources within the JD‐R model.

In summary, through the JD‐R model, we explored the impact of different types of career shocks on nurses’ different types of job performance and the moderating role of psychological resilience. The results not only guide nursing managers and policymakers in dealing with career shocks but also provide practical management strategies to boost nurses’ job performance in complex work settings, thus enhancing nursing teams’ service quality and healthcare organizations’ competitiveness.

## 2. Theoretical Basis and Research Hypotheses

### 2.1. Relationship Between Career Shocks and Nurses’ Job Performance

Career shocks significantly influence nurses’ job performance. Job performance serves as a dynamic representation of employees’ input, behaviors, processes, and outcomes. Additionally, it mirrors the objectives and duties that employees can fulfill [27]. In 1993, Borman and Motowidlo divided employee job performance into two principal aspects: task performance and contextual performance. Task performance centers on the execution of tasks in relation to the proficiency level of the employee and relates to their specific duties. This aspect of performance is often referred to as in‐role performance [28]. In contrast, contextual performance highlights the significance of nurturing positive workplace relationships, which can improve employees’ capacity to accomplish tasks more effectively. This type of performance emerges from behaviors that promote organizational development and go beyond employees’ typical responsibilities [29]. In specific nursing contexts, task performance focuses on nurses’ core professional duties, such as direct patient care, while contextual performance reflects teamwork and collaboration with physicians and other departments, including smooth shift handovers.

Scholars have identified that career shocks can be categorized as either positive or negative. As per the JD‐R model, positive career shocks may serve as a valuable job resource, as they can facilitate personal growth and development. Conversely, negative career shocks usually present obstructive demands, leading individuals to perceive a misalignment between their values and goals and their environment, thereby impeding their progress [30].

Positive career shocks often represent the affirmation and recognition of the individual, providing the individual with more resources, choices, and career opportunities and increasing the availability of other alternative career choices, which in turn increases the possibility of realizing career aspirations [30]. Mansur and Felix reported that positive career shocks significantly enhance an individual’s capacity to thrive [31].

However, the effect of negative career shocks cannot be ignored. Pak et al. examined employees over the age of 50 and reported that negative career shock events may affect the levels of job resources and job requirements to varying degrees [32]. Hofer et al. conducted a three‐wave quantitative study on 728 Swiss employees and reported that organization‐related negative career shock events can lead to increased job insecurity, which is subsequently associated with lower occupational optimism [33].

In summary, extensive literature and research findings show that career shocks significantly impact employees’ career development and job performance in both positive and negative ways. Therefore, the following hypotheses are proposed:

  Hypothesis 1: Positive career shocks positively predict (a) nurses’ task performance and (b) contextual performance.
 Hypothesis 2: Negative career shocks negatively predict (a) nurses’ task performance and (b) contextual performance.

### 2.2. The Moderating Effect of Psychological Resilience

Psychological resilience is the ability of an individual to achieve adaptive development in the face of adversity by mobilizing internal and external resources [34]. Some studies have shown that individuals with high levels of psychological resilience are more positive and optimistic and have good mental and emotional regulation abilities. At the same time, they can mobilize positive psychological resources to alleviate the negative influence of the outside world on the individual [34]. Following the JD‐R model, psychological resilience can be considered a valuable psychological resource [35]. It has a profound and positive effect on an individual’s work behavior and attitude.

When nurses are faced with positive career shocks, those with high psychological resilience are able to face these shocks with a more positive attitude and confidence. Consequently, this fosters a beneficial impact of such positive career shocks on their job performance. On the other hand, when nurses experience adverse career shocks, those who possess strong psychological resilience are more adept at managing their emotions and mental state, employing effective coping mechanisms to alleviate the stress inflicted by these negative career shocks. They may even perceive such stress not as a threat but as a challenge. Through their adaptability and capacity for recovery, individuals can swiftly recover from stress, thereby eroding the adverse effects of negative career shocks on their job performance. In summary, the following hypotheses are proposed:

 Hypothesis 3: Psychological resilience positively moderates the effect of positive career shocks on (a) nurses’ task performance and (b) contextual performance.
 Hypothesis 4: Psychological resilience negatively moderates the effect of negative career shocks on (a) nurses’ task performance and (b) contextual performance.

**FIGURE 1:**
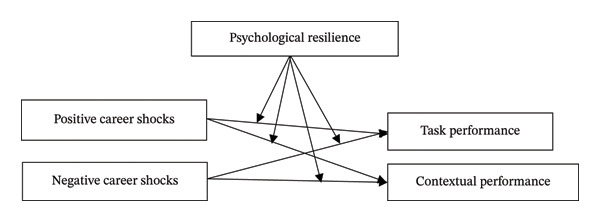
Theoretical framework.

To summarize, the model proposed in this paper is illustrated as follows, as depicted in Figure 1.

## 3. Methods

### 3.1. Participants and Procedure

According to the principle of estimating sample size (5–10 times the number of items), this study’s 41 items required 205–410 participants [36, 37]. After adjusting for an estimated 10% attrition rate during data collection, we set our target sample size at 225–450 and the minimum acceptable sample size at 205 to ensure the robustness of statistical analyses. Since structural equation modeling (SEM) requires a larger sample size [38], we initially distributed 450 questionnaires.

From March to May 2024, convenience sampling was employed via an online survey for registered nurses in general or tertiary hospitals in China. The inclusion criteria were as follows: (1) registered nurses in direct patient care and (2) over 6 months of clinical experience. The study was approved by the institutional ethics committee. Prior to participation, we provided a detailed electronic informed consent form explaining the research purpose, risks, and benefits. All participants provided written informed consent by clicking “Agree” before proceeding to the survey. Participants were informed of their right to withdraw at any time without penalty.

The data collection and cleaning followed a two‐step iterative process to ensure high data quality: Initial collection: 450 questionnaires were distributed. After removing responses with missing data (> 20%) or obvious careless patterns, 385 surveys were preliminarily eligible. However, a stricter logical consistency audit and outlier analysis reduced the strictly valid responses to 235. While meeting the minimum threshold, this size (5.7 times the items) was deemed insufficient for stable SEM estimation and raised concerns regarding discriminant validity. Supplemental collection and final merging: To enhance the measurement model’s validity and statistical power, an additional 200 questionnaires were collected following the same protocol. These were merged with the initial valid pool. Final analytic sample: A unified, stringent screening (addressing attention checks, patterned responses, and Mahalanobis distance for outliers) was applied to the entire combined pool. This resulted in a final high‐quality analytic sample of 385 participants (9.4 times the number of items). This final sample of 385 was used consistently across all demographic reporting (Table 1) and subsequent hypothesis testing.

**TABLE 1:** Demographic characteristics.

Demographics	Classification	Frequency	Percentage (%)
Gender	Male Female	116 269	30.13 69.87

Age	25 years old and below Between 26 and 35 Between 36 and 45 46 years old and above	58 166 105 56	15.06 43.12 27.27 14.55

Education	Specialized and below Undergraduate Master’s and above	100 155 60	31.80 49.20 19.00

Years of tenure	1 year and below Between 1 and 3 years Between 3 and 5 years 5 years and above	57 89 167 72	14.80 23.12 43.38 18.70

### 3.2. Measures

The scales employed in this study are recognized for their dependability and accuracy and are well established in the field. The items of the questionnaires are measured using a 5‐point Likert scale, where 1 represents “*strongly disagree*” and 5 represents “*strongly agree*.” The measurement of Cronbach’s alpha falls under reliability analysis. Nunnally indicates that Cronbach’s alpha greater than 0.7 meets the required standard [39].

#### 3.2.1. Career Shocks

We measured career shocks adapted from the scale used by Mansur and Felix [31]. The six items were based on the work of Seibert et al. [14] and Holtom et al.[40]. An example of a positive shock was “Received an unexpectedly better job offer,” whereas a negative shock might be described as “I missed an opportunity for promotion.” Cronbach’s α coefficients of the scales in this paper were 0.856 and 0.829, respectively.

#### 3.2.2. Job Performance

Employees reported their job performance via the scale developed by Borman and Motowidlo and translated into Chinese by scholar Yu [41]. This scale encompasses two dimensions: contextual performance and task performance, including a total of 10 items. Examples include “My work can meet the expected standards” and “I frequently cooperate with other colleagues.” Cronbach’s α coefficients of the scale in this paper were 0.826 and 0.830, respectively.

#### 3.2.3. Psychological Resilience

The measure of psychological resilience was developed by Yu [42]. The scale contains three dimensions, “strength,” “optimism,” and “tenacity,” with a total of 25 items. The items included “I can adapt to change” and “I have a close relationship.” Cronbach’s α coefficient of the scale in this study was 0.912.

The reliability of all five scales in this study was well above the generally accepted threshold value of 0.70.

#### 3.2.4. Control Variables

Earlier research indicates that demographic factors such as gender and tenure can influence career shocks, psychological resilience, and job performance [25]. Consequently, we incorporated these factors as control variables. In accordance with the current literature [4], all aforementioned variables were transformed into dummy variables prior to the data analysis. For instance, gender was coded as a dummy variable (0 = female, 1 = male).

### 3.3. Ethics Statement

Formal approval was obtained from the Ethics Committee for Biomedical Research at the Medical College of Hebei University of Engineering (approval number: 2023[K]030–20) before the initiation of the survey. Data privacy and confidentiality were maintained and assured by obtaining participants’ informed consent to participate in the research. All participants provided written informed consent for participation. The informed consent form is attached in the appendix for details.

## 4. Results

### 4.1. Common Method Bias Testing

Because the data in this study were self‐reported and derived from a single source, we implemented Harman’s single‐factor test to manage common method variance. The findings indicated that the variance accounted for by the initial principal component analysis was 30.639% (less than 40%), suggesting that common method bias was not significant [43].

### 4.2. Confirmatory Factor Analysis (CFA)

CFA pertains to validity analysis. Following Anderson [44], CFA was performed to estimate the factor loading of each construct and the measurement model fit. For this study, Mplus 8.0 was utilized to analyze the sample data, and a competitive model incorporating five essential variables—positive/negative career shocks, psychological resilience, and task/contextual performance—was developed to evaluate the model’s validity. As presented in Table 2, the five‐factor model (positive/negative career shocks, psychological resilience, and task/contextual performance) had the best overall fitting validity (*χ* ^2^/df = 1.78 < 3; CFI = 0.903 > 0.900; TLI = 0.897 > 0.800; RMSEA = 0.045 < 0.08; SRMR = 0.058 < 0.08). The above measured values are all within the acceptable range, indicating that the proposed model fit the data well and was suitable for testing the research hypotheses [45, 46]. It also indicated that the above variables have good discriminant validity.

**TABLE 2:** Results of the confirmatory factor analysis.

Fit indicators	*χ* ^2^	df	*χ* ^2^/df	CFI	TLI	RMSEA	SRMR
Five‐factor model	1372.245	769	1.78	0.903	0.897	0.045	0.058
Four‐factor model	1393.046	773	1.80	0.901	0.895	0.046	0.061
Three‐factor model	1409.317	776	1.81	0.898	0.893	0.046	0.061
Two‐factor model	2075.939	778	2.67	0.792	0.781	0.066	0.085
One‐factor model	2365.247	779	3.04	0.746	0.732	0.073	0.087

*Note:* One‐factor model = PCS + NCS + PR + TP + CP; two‐factor model = PCS + NCS, PR + TP + CP; three‐factor model = PCS + NCS, PR, TP + CP; four‐factor model = PCS + NCS, PR, TP, CP; five‐factor model = PCS, NCS, PR, TP, CP.

Abbreviations: CP = contextual performance, NCS = negative career shocks, PCS = positive career shocks, PR = psychological resilience, TP = task performance.

### 4.3. Correlation Analysis

Table 3 displays the descriptive statistics and the correlations between the variables. This shows that the correlation between the variables in this study was good, which laid the foundation for the testing of the hypotheses in subsequent research.

**TABLE 3:** Means, standard deviations, and correlations of the variables.

Variables	M	SD	1	2	3	4	5	6	7	8	9
1. Gender	1.54	0.499	1								
2. Education	2.25	0.877	0.115^∗^	1							
3. Age	2.22	0.964	0.010	0.028	1						
4. Tenure	2.06	0.808	0.033	−0.127^∗^	0.027	1					
5. PCS	3.07	0.855	−0.059	−0.061	0.043	0.013	1				
6. NCS	3.16	0.757	−0.047	−0.025	0.103^∗^	0.034	0.436^∗∗^	1			
7. PR	3.39	0.821	−0.011	−0.085	0.059	0.044	0.147^∗∗^	0.220^∗∗^	1		
8. TP	3.13	0.904	0.033	−0.024	0.065	−0.032	0.156^∗∗^	0.191^∗∗^	0.256^∗∗^	1	
9. CP	3.26	0.832	0.003	0.037	0.054	−0.059	0.133^∗∗^	0.177^∗∗^	0.280^∗∗^	0.611^∗∗^	1

*Note: N* = 385, two‐sided test.

Abbreviations: CP = contextual performance, NCS = negative career shocks, PCS = positive career shocks, PR = psychological resilience, TP = task performance.

^∗^ *p* < 0.05.

^∗∗^ *p* < 0.01.

### 4.4. Hypothesis Testing

**FIGURE 2:**
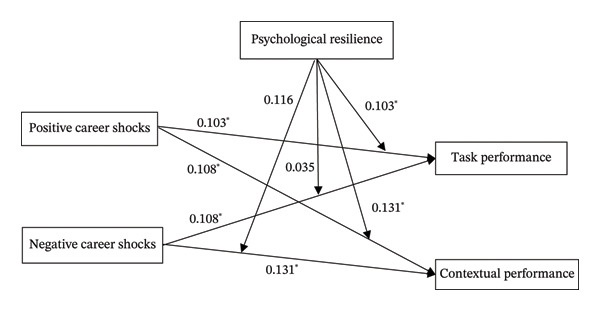
Moderating effect of psychological resilience on career shocks and job performance among nurses. ^∗^ *p* < 0.05.

We utilized Mplus 8.0 for SEM to test our model. The detailed results of the SEM are provided in Figure 2. After considering the control variables, the primary effects were confirmed for both paths. As shown in Figure 2, positive career shocks could positively predict nurses’ task performance (*b* = 0.103, *p* < 0.05) as well as contextual performance (*b* = 0.108, *p* < 0.05). Therefore, H1a and H1b were verified. Negative career shocks could positively predict task performance (*b*1 = 0.108, *p* < 0.05) and contextual performance (*b*2 = 0.131, *p* < 0.05). Therefore, the findings for negative career shocks were opposite to the hypothesized directions, and H2a and H2b were not supported.

As illustrated in Figure 2, the interaction between positive career shocks and psychological resilience showed a significantly positive relationship with both task performance (*b* = 0.103, *p* < 0.05) and contextual performance (*b* = 0.131, *p* < 0.05) among nurses. Therefore, H3a and H3b were affirmed. Furthermore, the interaction term of negative career shocks and psychological resilience did not significantly impact nurses’ task performance (*b* = 0.035, *p* = 0.586) and contextual performance (*b* = 0.116, *p* = 0.185). Therefore, H4a and H4b were not supported.

**FIGURE 3:**
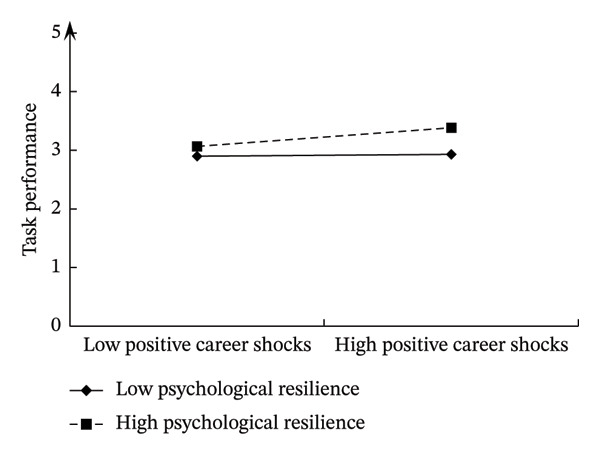
Moderating effect of psychological resilience on positive career shocks and task performance.

**FIGURE 4:**
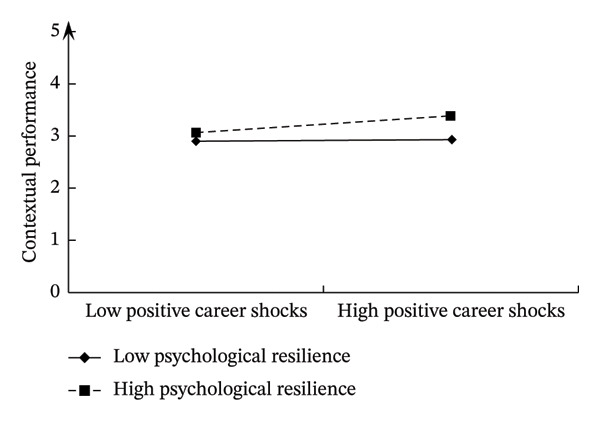
Moderating effect of psychological resilience on positive career shocks and contextual performance.

This study drew moderating effect diagrams to show the variable relationships and interaction effects more intuitively. The details are shown in Figures 3 and 4. When psychological resilience was low, positive career shocks positively impacted nurses’ task performance (*b* = 0.369, *p* < 0.01) and contextual performance (*b* = 0.444, *p* < 0.01). Conversely, with high psychological resilience, positive career shocks positively influenced both task performance (*b* = 0.538, *p* < 0.01) and contextual performance (*b* = 0.658, *p* < 0.01).

## 5. Discussion

### 5.1. Interpreting the Findings

First, positive career shocks have a positive relationship with nurses’ job performance in both task and contextual aspects. This observation is consistent with previously established research findings. Prior studies have typically linked positive career shocks to favorable outcomes [4, 33]. Mehreen noted that, functioning as a type of emotional trigger, career shocks provoked either positive or negative emotional reactions, which in turn influenced behavioral adjustments in individuals [25].

Second, negative career shocks positively predict both nurses’ task performance and contextual performance. Most studies portray negative career shocks as precursors to pessimistic attitudes and detrimental behaviors [31]. However, a nascent strand of research has begun to uncover their potential upside—reframing negative career shocks as opportunities for growth [47], launching new career paths [48] or motivating proactive resource acquisition [49]. This finding also responds to scholars’ calls to examine the positive effects of external negative events on proactive behavior [50, 51]. Notably, Akkermans et al. pointed out that the short‐term and long‐term effects of career shocks may differ substantially [15]. Given that this study adopted a cross‐sectional design, it is difficult to reveal long‐term effects. Therefore, the findings are somewhat limited and should be regarded as preliminary only. Future research may adopt a longitudinal design to further explore the long‐term effects.

In addition, the positive effect of negative career shocks on nurses’ job performance may be attributed to the special adaptive mechanisms inherent in the nursing profession within China’s healthcare system. Under the dual influence of the “reverencing life and healing the wounded” value orientation in Chinese nursing culture and the professional ethical expectation of “willingness to dedication” within a collectivist context, nurses are more likely to experience posttraumatic growth (PTG) when facing adverse career events such as doctor–patient conflicts, occupational injuries, medical errors, critical resuscitation situations, and department transfers. PTG refers to the psychological process through which individuals achieve enhanced psychological resilience and personal growth via positive cognitive restructuring and adaptive coping strategies following traumatic events, characterized by a reevaluation of self‐efficacy, deep reflection on professional values, and active construction of coping resources [52]. Thus, these negative events promote nurses’ professional growth, thereby positively influencing their job performance.

Furthermore, psychological resilience demonstrated a significant positive moderating effect on the relationship between positive career shocks and nurses’ job performance. As indicated by the JD‐R model, positive career shocks can be conceptualized as resources that promote personal growth and development, thereby enhancing the job performance of nurses [53]. When nurses possess a high level of psychological resilience, they are better equipped to approach these shocks with a constructive mindset and self‐assurance, thereby enhancing the favorable effects of positive career shocks on their job performance.

Moreover, the moderating effect of psychological resilience between negative career shocks and nurses’ job performance was not supported. Although negative career shocks may produce positive behaviors and attitudes, they are essentially negative emotional events that trigger individuals’ adverse feelings such as anger, frustration, and helplessness [2]. Moreover, nurses in China are routinely exposed to multiple stressors: heavy patient loads, the emotional strain of caring for the dying, high expectations from patients’ relatives, continuous upgrades in medical technology, and inadequate support from peers and supervisors. Consequently, they must sustain a high level of effort simply to meet everyday job demands [4, 54].

From the perspective of COR theory, nurses high in psychological resilience are likely to invest additional resources (e.g., time, energy, and psychological resources) to cope with negative career shocks. However, when the intensity or duration of the shock exceeds one’s threshold, even highly resilient individuals can experience resource depletion and emotional exhaustion [55]. Under such conditions, resilience cannot enhance job performance; at best, it helps the nurse maintain baseline job performance.

Finally, both positive and negative career shocks demonstrated a significantly stronger positive association with contextual performance than with task performance. This asymmetric effect stems from the essential differences between the two performance dimensions in terms of resource mobilization flexibility and institutional constraint intensity. It is further amplified by specific cultural logics within the Chinese nursing context. Specifically, task performance, as behavior directly serving the technical core of nursing, is subject to rigid constraints from strict operational protocols, regulatory requirements, and job descriptions, rendering it relatively stable [56], whereas contextual performance, as spontaneous behavior maintaining the sociopsychological environment, possesses high strategic flexibility and substitutability [29].

Furthermore, within the unique context of Chinese indigenous culture characterized by collectivism and an emphasis on interpersonal face, this dynamic is particularly pronounced [57, 58]. Specifically, when facing positive shocks, nurses actively engage in collaboration and information sharing to “gain face,” thereby consolidating their newly acquired recognition and status; when confronting negative shocks, nurses strengthen cooperation and maintain harmony to “save face,” avoiding the embarrassment and isolation resulting from career setbacks. This sensitivity to and cultivation of face resources make contextual performance the preferred strategy for nurses to cope with career fluctuations—it allows them to quickly demonstrate value and garner support while being more adjustable and interpersonally rewarding than task performance. Consequently, career shocks activate the prioritized mobilization of face and relational resources, thereby exerting a more pronounced positive driving effect on contextual performance in the Chinese nursing context.

### 5.2. Contributions to the Literature

First, this paper echoes the call from prior studies to explore how environmental factors (such as career shocks) affect career development [24, 59, 60]. In addition, this paper also studies the effect of career shocks on the job performance of nurses, which answers the call of scholars [4].

Second, this research direction is in line with the suggestions of previous scholars, who advocated studying these two types of career shocks simultaneously [25]. The detailed study of these two types of career shocks is helpful for obtaining a more in‐depth understanding of the specific manifestation and action mechanism of career shocks in nursing management practice.

Finally, the present study expands the study on the boundary conditions of career shocks by introducing psychological resilience as a moderating variable. It also systematically responds to the latest academic initiatives of scholars to further investigate the moderating effect of personnel characteristics in the process of coping with career shocks [4]. Moreover, by organically embedding psychological resilience as a psychological resource in the JD‐R model, this study not only verified psychological resilience as a core psychological resource in the context of career shocks but also expanded the application of the JD‐R model from the theoretical level.

### 5.3. Implications for Nursing Management

Our study proposes layered management strategies for nurse managers, nursing leaders, and human resources departments to establish a systematic intervention mechanism, providing a theoretical foundation and practical insights for addressing career shocks and improving nurses’ job performance.

First, nurse managers and frontline nursing supervisors should pay closer attention to various unexpected career shocks that may occur in nurses’ daily work. They should also carry out specialized training programs on emotional management to help nurses effectively cope with the adverse effects of external career shocks. Meanwhile, managers should consistently provide support for nurses to improve their self‐efficacy and psychological resilience, strengthening their ability to address professional challenges.

Second, studies have indicated that the organizational climate and leaders’ support can enhance employees’ psychological resilience [61, 62]. Thus, nursing leaders should cultivate a supportive organizational culture and practice environment to boost nurses’ confidence and competence in dealing with career shocks. At the same time, nursing leaders ought to persist in providing support that boosts employees’ organizational trust and self‐efficacy, ultimately improving their ability to address challenges.

Third, the human resources department should actively promote the development of a psychological resilience cultivation system for nursing students, such as systematically integrating mindfulness‐based stress reduction and emotion regulation training into the core curriculum of nursing education and constructing a three‐dimensional support network featuring “professional mentorship by clinical instructors, peer mutual emotional support, and precise psychological counseling intervention” to enhance nursing students’ psychological adaptability in coping with academic stress and professional challenges from multiple dimensions, thereby laying a solid psychological foundation for their future transition into clinical positions and enabling them to calmly navigate career shocks.

### 5.4. Limitations and Future Research

This study has several limitations in data collection. First, the cross‐sectional design makes it difficult to establish causal relationships among variables and fails to capture their dynamic evolution. To address this limitation, future research should adopt longitudinal designs to track the dynamic relationships among variables, thereby enhancing the reliability of causal inferences. Second, this study employed self‐report measures, which may be subject to recall bias and social desirability effects; single‐source data may introduce common method bias, which cannot be completely eliminated despite control measures such as randomizing questionnaire items. Thus, future research should incorporate objective indicators and other‐rated data to validate self‐reported results, thereby improving the accuracy and objectivity of the findings. Third, the contextual details are limited, as all samples in this study were from Chinese nurses, and the regional and institutional characteristics of the sample may restrict the generalizability of the results. Future research should conduct cross‐cultural comparative studies in different contexts, thereby enhancing the external validity of research conclusions.

Furthermore, the present study only considered psychological resilience as a moderator and did not explore the underlying psychological mechanisms. In actual work settings, nurses’ perceptions and reactions to career shocks can be intricate and variable. These responses are influenced not only by psychological resilience but may also be moderated by additional factors such as social support [19] or mediated by other variables such as self‐efficacy. Therefore, future research should incorporate a broader range of moderating and mediating variables to better uncover the mechanisms through which career shocks affect nurses’ job performance and to help nurses cope more effectively with such shocks.

Finally, this study focused exclusively on nurses. Qian et al. note that industries and positions differ in their characteristics, so the manifestations of career shocks also vary by context [63]. Future research should therefore disaggregate the nursing sample by specialty and role to enhance generalizability, pinpoint the key buffering factors within distinct care settings, and furnish hospital managers with evidence for tailored support strategies.

## 6. Conclusion

This study has revealed that both positive and negative career shocks positively predict nurses’ job performance. Psychological resilience exerted a significant positive moderating effect on the association between positive career shocks and nurses’ job performance, amplifying the beneficial effects of positive shocks. The findings offer a theoretical foundation and practical insights for nursing managers and policymakers to handle career shocks and boost nurses’ job performance.

## Funding

This work was funded by the National Natural Science Foundation of China Project (72471027).

## Conflicts of Interest

The authors declare no conflicts of interest.

## Data Availability

The data that support the findings of this study are available from the corresponding author upon reasonable request.

## References

[1] Bennett N. and Lemoine G. J. , What a Difference a Word Makes: Understanding Threats to Performance in a VUCA World, Business Horizons. (2014) 57, no. 3, 311–317, 10.1016/j.bushor.2014.01.001, 2-s2.0-84898775367.

[2] Feng J. , Jiang X. , and Zhou W. , Career Shocks: Conceptualizations, Measurements, Antecedents and Consequences, Human Resources Development of China. (2021) 38, no. 5, 6–24.

[3] Ma Y. , Bennett D. , and Donald W. E. , Navigating the Storm: Understanding the Impact of a COVID-19-induced Negative Career Shock on Career Success and Life Satisfaction in China, Journal of Work-Applied Management. (2025) .

[4] Zhang Y. , Bu X. , and Zhang N. , Increasing Nurses’ Occupational well-being: The Role of Career Shocks, Job Crafting and Supervisor Autonomy Support, BMC Nursing. (2024) 23, no. 1, 1–8, 10.1186/s12912-024-01955-4. 38679701 PMC11056045

[5] Zhang Z. , Yang C. , Wang Y. , Deng G. , and Chang J. , Investigating the Intentions and Reasons of Senior High School Students in Registering for Nursing Education in China, BMC Nursing. (2023) 22, no. 1, 10.1186/s12912-023-01480-w. PMC10496206 37700328

[6] Hu H. , Wang C. , Lan Y. , and Wu X. , Nurses’ Turnover Intention, Hope and Career Identity: The Mediating Role of Job Satisfaction, BMC Nursing. (2022) 21, no. 1, 10.1186/s12912-022-00821-5. PMC8830989 35144604

[7] Greenhaus J. H. , Callanan G. H. , and Godshalk V. M. , Career Management (4Thed), 2010, Sage Publications.

[8] Lent R., W. , Brown S., & D. , and Hackett G. , Toward a Unifying Social Cognitivetheory of Career and Academic Interest, Choice, and Performance, Journal of Vocational Behavior. (1994) 45, no. 1, 79–122, 10.1006/jvbe.1994.1027, 2-s2.0-43949159942.

[9] Zhang N. , Xu D. , Li J. , and Xu Z. , Effects of Role Overload, Work Engagement and Perceived Organisational Support on Nurses’ Job Performance During the COVID-19 Pandemic, Journal of Nursing Management. (2022) 30, no. 4, 901–912, 10.1111/jonm.13598. 35293044 PMC9115180

[10] Akkermans J. , Seibert S. E. , and Mol S. T. , Tales of the Unexpected: Integrating Career Shocks in the Contemporary Careers Literature, SA Journal of Industrial Psychology. (2018) 44, no. 1, 1–10, 10.4102/sajip.v44i0.1503, 2-s2.0-85046783959.

[11] Akkermans J. , Collings D. G. , da Motta Veiga S. P. , Post C. , and Seibert S. , Toward a Broader Understanding of Career Shocks: Exploring Interdisciplinary Connections with Research on Job Search, Human Resource Management, Entrepreneurship, and Diversity, Journal of Vocational Behavior. (2021) 126, 10.1016/j.jvb.2021.103563.

[12] Holtom B. C. , Burton J. P. , and Crossley C. D. , How Negative Affectivity Moderates the Relationship Between Shocks, Embeddedness and Worker Behaviors, Journal of Vocational Behavior. (2012) 80, no. 2, 434–443, 10.1016/j.jvb.2011.12.006, 2-s2.0-84857789682.

[13] Nery-Kjerfve T. and Wang J. , Transfer from Expatriate to Local Contracts: A Multiple Case Study of an Unexpected Career Transition, Human Resource Development International. (2019) 22, no. 3, 235–256, 10.1080/13678868.2019.1570776, 2-s2.0-85061437174.

[14] Seibert S. E. , Kraimer M. L. , Holtom B. C. , and Pierotti A. J. , Even the Best Laid Plans Sometimes Go Askew: Career Self-Management Processes, Career Shocks, and the Decision to Pursue Graduate Education, Journal of Applied Psychology. (2013) 98, no. 1, 169–182, 10.1037/a0030882, 2-s2.0-84877048161. 23181345

[15] Akkermans J. , Richardson J. , and Kraimer M. L. , The Covid-19 Crisis as a Career Shock: Implications for Careers and Vocational Behavior, Journal of Vocational Behavior. (2020) 119, 10.1016/j.jvb.2020.103434. PMC7205633 32390655

[16] Cheraghi R. , Parizad N. , Alinejad V. , Piran M. , and Almasi L. , The Effect of Emotional Intelligence on Nurses’ Job Performance: The Mediating Role of Moral Intelligence and Occupational Stress, BMC Nursing. (2025) 24, no. 1, 10.1186/s12912-025-02744-3. PMC11796207 39905367

[17] Zhang N. , Liu X. , and Jin C. , Effects of STARA Awareness on the Job Performance of Healthcare Providers: The Mediating Role of Qualitative Job Insecurity, International Journal of Internet Manufacturing and Services. (2025) 11, no. 2, 171–189, 10.1504/ijims.2025.146821.

[18] Hennekam S. , Ladge J. J. , and Powell G. N. , Confinement During the COVID-19 Pandemic: How Multi Domain work-life Shock Events May Result in Positive Identity Change, Journal of Vocational Behavior. (2021) 130, 10.1016/j.jvb.2021.103621. PMC8416426 34511627

[19] Wordsworth R. and Nilakant V. , Unexpected Change: Career Transitions Following a Significant Extra-organizational Shock, Journal of Vocational Behavior. (2021) 127, 10.1016/j.jvb.2021.103555.

[20] Grover S. L. , Teo S. T. T. , Pick D. , Roche M. , and Newton C. J. , Psychological Capital as a Personal Resource in the JD-R Model, Personnel Review. (2018) 47, no. 4, 968–984, 10.1108/pr-08-2016-0213, 2-s2.0-85046796012.

[21] Lussier I. , Derevensky J. L. , Gupta R. , Bergevin T. , and Ellenbogen S. , Youth Gambling Behaviors: An Examination of the Role of Resilience, Psychology of Addictive Behaviors. (2007) 21, no. 2, 165–173, 10.1037/0893-164x.21.2.165, 2-s2.0-34548860975. 17563136

[22] Hu T. , Zhang D. , and Wang J. , A meta-analysis of the Trait Resilience and Mental Health, Personality and Individual Differences. (2015) 76, 18–27, 10.1016/j.paid.2014.11.039, 2-s2.0-84949114846.

[23] Burton J., P. , Holtom , Brooks C. , and Thomas W. , The Buffering Effects of Job Embeddedness on Negative Shocks, Journal of Vocational Behavior. (2009) 76, no. 1, 42–51.

[24] Nalis I. , Kubicek B. , and Korunka C. , From Shock to Shift-A Qualitative Analysis of Accounts in Mid-Career About Changes in the Career Path, Frontiers in Psychology. (2021) 12, 10.3389/fpsyg.2021.641248. PMC7952510 33716910

[25] Mehreen A. and Ali Z. , Really Shocks Cannot be Ignored: The Effects of Career Shocks on Career Development and How Family Support Moderates This Relationship?, International Journal for Educational and Vocational Guidance. (2024) 24, no. 3, 701–726, 10.1007/s10775-022-09574-8.

[26] Canale N. , Marino C. , Griffiths M. D. , Scacchi L. , Monaci M. G. , and Vieno A. , The Association Between Problematic Online Gaming and Perceived Stress: The Moderating Effect of Psychological Resilience, Journal of Behavioral Addictions. (2019) 8, 1–7, 10.1556/2006.8.2019.01, 2-s2.0-85064003038. 30739461 PMC7044594

[27] Murphy K. R. , Schmitt N. , and Borman W. C. , Personnel Selection in Organizations, Academy of Management Review. (1993) 18, no. 4, 783–785, 10.2307/258598.

[28] Schmidt F. L. , Hunter J. E. , and Outerbridge A. N. , Impact of Job Experience and Ability on Job Knowledge, Work Sample Performance, and Supervisory Ratings of Job Performance, Journal of Applied Psychology. (1986) 71, no. 3, 432–439, 10.1037/0021-9010.71.3.432, 2-s2.0-58149372734.

[29] Scotter J. R. V. and Motowidlo S. J. , Interpersonal Facilitation and Job Dedication as Separate Facets of Contextual Performance, Journal of Applied Psychology. (1996) 81, no. 5, 525–531, 10.1037/0021-9010.81.5.525, 2-s2.0-0030505850.

[30] Kraimer M. L. , Greco L. , Seibert S. E. , and Sargent L. D. , An Investigation of Academic Career Success: The New Tempo of Academic Life, The Academy of Management Learning and Education. (2019) 18, no. 2, 128–152, 10.5465/amle.2017.0391, 2-s2.0-85070364112.

[31] Mansur J. and Felix B. , On Lemons and Lemonade: The Effect of Positive and Negative Career Shocks on Thriving, Career Development International. (2021) 26, no. 4, 495–513, 10.1108/cdi-12-2018-0300.

[32] Pak K. , Kooij D. , Lange A. H. D. , Meyers M. C. , and Veldhoven M. V. , Unravelling the Process Between Career Shock and Career (Un)Sustainability: Exploring the Role of Perceived Human Resource Management, Career Development International. (2021) 26, no. 4, 514–539, 10.1108/cdi-10-2018-0271.

[33] Hofer A. , Spurk D. , and Hirschi A. , When and Why do Negative Organization-Related Career Shocks Impair Career Optimism? A Conditional Indirect Effect Model, Career Development International. (2021) 26, no. 4, 467–494, 10.1108/cdi-12-2018-0299.

[34] Masten A. S. , Lucke C. M. , Nelson K. M. , and Stallworthy I. C. , Resilience in Development and Psychopathology: Multisystem Perspectives, Annual Review of Clinical Psychology. (2021) 17, no. 1, 521–549, 10.1146/annurev-clinpsy-081219-120307. 33534615

[35] Xanthopoulou D. , Bakker A. B. , Demerouti E. , and Schaufeli W. B. , The Role of Personality in the Job Demands‐Resources Model, International Journal of Stress Managment. (2007) 14, no. 2, 121–141.

[36] Bentler P. M. and Chou C. P. , Practical Issues in Structural Modeling, Sociological Methods & Research. (1987) 16, no. 1, 78–117, 10.1177/0049124187016001004, 2-s2.0-84970378038.

[37] Gorsuch R. L. , Three Methods for Analyzing Limited time-series (N of 1) Data, Behavioral Assessment. (1983) 7, no. 2, 141–154.

[38] Schumacker R. E. and Lomax R. G. , A Beginner’s Guide to Structural Equation Modeling, 2010, Routledge.

[39] Nunally J. C. , Psychometric Theory, 1978, McGraw-Hill, New York.

[40] Holtom B. C. , Mitchell T. R. , Lee T. W. , and Inderrieden E. J. , Shocks as Causes of Turnover: What They are and How Organizations Can Manage Them, Human Resource Management. (2005) 44, no. 3, 337–352, 10.1002/hrm.20074, 2-s2.0-25444511336.

[41] Yu D. , The Impact of Quality Management Human Face System Factors on Job Performance, 1996, National Sun Yat-sen University.

[42] Yu X. N. and Zhang J. , Factor Analysis and Psychometric Evaluation of the Connor-Davidson Resilience Scale (CD-RISC) with Chinese People, Social Behavior and Personality. (2007) 35, no. 1, 19–30, 10.2224/sbp.2007.35.1.19, 2-s2.0-33847706464.

[43] Hao Z. and Lirong L. , Statistical Remedies for Common Method Biases, Advances in Psychological Science. (2004) .

[44] Anderson J. , Gerbing C. , and David & W. , Structural Equation Modeling in Practice: A Review and Recommended Two-step Approach, Psychological Bulletin. (1988) 103, no. 3, 411–423, 10.1037/0033-2909.103.3.411, 2-s2.0-41649112685.

[45] Bagozzi R. P. and Yi Y. , On the Evaluation of Structural Equation Models, Journal of the Academy of Marketing Science. (1988) 16, no. 1, 74–94, 10.1007/bf02723327, 2-s2.0-51249177591.

[46] Bentler P. , Bonett M. , and Douglas & G. , Significance Tests and Goodness of Fit in the Analysis of Covariance Structures, Psychological Bulletin. (1980) 88, no. 3, 588–606, 10.1037/0033-2909.88.3.588, 2-s2.0-4243159210.

[47] Peeters E. R. , Caniëls M. C. , and Verbruggen M. , Dust Yourself off and Try Again: The Positive Process of Career Changes or Shocks and Career Resilience, Career Development International. (2022) 27, no. 3, 372–390, 10.1108/cdi-06-2021-0143.

[48] Baruch Y. and Lavi-Steiner O. , The Career Impact of Management Education from an average-ranked University: Human Capital Perspective, Career Development International. (2015) 20, no. 3, 218–237, 10.1108/cdi-08-2014-0117, 2-s2.0-84929734039.

[49] Feng J. , Zhou W. , Li S. , and Li M. , Obstacles Open the Door—Negative Shocks Can Motivate Individuals to Focus on Opportunities, Frontiers of Business Research in China. (2019) 13, no. 21, 10.1186/s11782-019-0067-9.

[50] Bindl U. K. , Work-Related Proactivity Through the Lens of Narrative: Investigating Emotional Journeys in the Process of Making Things Happen, Human Relations. (2019) 72, no. 4, 615–645, 10.1177/0018726718778086, 2-s2.0-85049039805.

[51] Lebel R. D. , Overcoming the Fear Factor: How Perceptions of Supervisor Openness Lead Employees to Speak up when Fearing External Threat, Organizational Behavior and Human Decision Processes. (2016) 135, 10–21, 10.1016/j.obhdp.2016.05.001, 2-s2.0-84969203952.

[52] Dell’Osso L. , Lorenzi P. , Nardi B. , Carmassi C. , and Carpita B. , Post Traumatic Growth (PTG) in the Frame of Traumatic Experiences, Clinical Neuropsychiatry. (2022) 19, no. 6, 390–393, 10.36131/cnfioritieditore20220606. 36627947 PMC9807114

[53] Demerouti E. and Bakker A. B. , Job demands-resources Theory in Times of Crises: New Propositions, Organizational Psychology Review. (2023) 13, no. 3, 209–236, 10.1177/20413866221135022.

[54] Dasgupta P. , Effect of Role Ambiguity, Conflict and Overload in Private Hospitals’ Nurses’ Burnout and Mediation Through Self Efficacy, Journal of Health Management. (2012) 14, no. 4, 513–534, 10.1177/0972063412468980, 2-s2.0-84872834389.

[55] Hobfoll S. E. , Halbesleben J. , Neveu J. P. , and Westman M. , Conservation of Resources in the Organizational Context: The Reality of Resources and Their Consequences, Annual Review of Organizational Psychology and Organizational Behavior. (2018) 5, no. 1, 103–128, 10.1146/annurev-orgpsych-032117-104640, 2-s2.0-85042677566.

[56] Stanton J. M. and Sarkar-Barney S. T. M. , A Detailed Analysis of Task Performance with and Without Computer Monitoring, International Journal of Human-Computer Interaction. (2003) 16, no. 2, 345–366, 10.1207/s15327590ijhc1602_11, 2-s2.0-0345377757.

[57] Hofstede G. , Culture’s Consequences: International Differences in work-related Values, 1984, Sage.

[58] Hwang K.-k. , Face and Favor: The Chinese Power Game, American Journal of Sociology. (1987) 92, no. 4, 944–974, 10.1086/228588.

[59] Akkermans J. and Kubasch S. , Trending Topics in Careers: A Review and Future Research Agenda, Career Development International. (2017) 22, no. 6, 586–627, 10.1108/cdi-08-2017-0143, 2-s2.0-85031813074.

[60] Kostal J. W. , Wiernik B. M. M. , and Viswesvaran C. , A meta-analytic Investigation of Demographic Differences in Protean, Boundaryless, and Proactive Career Orientations, Career Development International. (2017) 22, no. 5, 520–545, 10.1108/cdi-08-2017-0139, 2-s2.0-85030857548.

[61] Fan W. , Luo Y. , Cai Y. , and Meng H. , Crossover Effects of Leader’s Resilience: A Multilevel Mediation Approach, Journal of Managerial Psychology. (2020) 35, no. 5, 375–389, 10.1108/jmp-02-2019-0109.

[62] Salari F. , Investigation the Relationship Between Organizational Climate and Psychological Hardiness with Job Burnout of Personnel in University of Bandar abbas, Arth Prabandh: A Journal of Economics and Management. (2014) 3, no. 9, 155–164.

[63] Qian Z. , Chao M. A. , and Shuiping J. , The Role of Shocks in Employee Voluntary Turnover, Advances in Psychological Science. (2010) 18, no. 10, 1606–1611.

